# Sleep Hypoxemia as a Predictor of Mortality in Patients with Sleep Apnea: A Secondary Analysis of the Sleep Heart Health Study

**DOI:** 10.1016/j.chpulm.2024.100087

**Published:** 2024-07-23

**Authors:** Mohammad Masoudian Khouzani, Jack Botros, Mariela Padilla, Richard J. Castriotta

**Affiliations:** a Advanced Education in Orofacial Pain, Orofacial Pain and Oral Medicine Center, Herman Ostrow School of Dentistry, University of Southern California, Los Angeles, CA; b Herman Ostrow School of Dentistry, University of Southern California, Los Angeles, CA; c Division of Pulmonary, Critical Care and Sleep Medicine, Keck School of Medicine, University of Southern California, Los Angeles, CA

**Keywords:** apnea-hypopnea index, hypoxemia, mortality, oxygen saturation, sleep apnea

## Abstract

**Background:**

The Sleep Heart Health Study (SHHS) was a prospective cohort study formulated to explore the risk factors for development of cardiovascular disease in OSA, diagnosed via unaccompanied home sleep apnea test. We used these data to compare the association of the apnea-hypopnea index (AHI) and amount of sleep hypoxemia with the risk of all-cause mortality.

**Research Question:**

What is the relationship among hypoxemia, AHI, and mortality in OSA?

**Study Design and Methods:**

We compared the association of (1) the AHI, (2) the percentage of sleep time with oxygen saturation < 85% (PERC85), and (3) the duration of sleep (in minutes) with oxygen saturation < 85% (MIN85) with the risk of all-cause mortality in the SHHS. Multivariable logistic regression analyses were used and adjusted for age, sex, BMI, pack-years of smoking, cardiovascular score at baseline, and treatment status.

**Results:**

PERC85 was associated with an increased risk of death (OR, 1.03; 95% CI, 1.01-1.05; *P* = .003). Patients with PERC85 of 1% to 5%, 5% to 20%, and > 20% showed progressively higher risks compared with those with PERC85 of < 1% (1%-5%: OR, 1.37 [95% CI, 1.02-1.83]; 5%-20%: OR, 1.76 [95% CI, 1.07-2.86]; > 20%: OR, 2.93 [95% CI, 1.20-6.98]; *P < .*05 for all). The MIN85 predicted all-cause mortality (OR, 1.01 [95% CI, 1.00-1.01]; *P = .*009). Participants with 2 to 30 min and > 30 min of PERC85 showed higher likelihoods of death vs those with PERC85 of < 2 min (2-30 mins: OR, 1.29 [95% CI, 1.01-1.63]; > 30 min: OR, 2.15 [95% CI, 1.22-3.76]; *P* < .05 for all). AHI was not associated with an increased risk of mortality.

**Interpretation:**

Our findings indicate that sleep hypoxemia with MIN85 is a better predictor of mortality in OSA than AHI. Monitoring oxygen saturation levels and duration may be important for risk stratification and assessment of treatment adequacy in OSA, although this may be confounded by hypoxemia not related to OSA.

**Take-home Points**

**Study Question:** Which parameters of sleep studies are most important for mortality risk in OSA?

**Results:** In this study, sleep hypoxemia with oxygen saturation time < 85% correlated best with mortality, whereas apnea-hypopnea index showed no significant mortality correlation in itself. Nontreatment of OSA also was associated with mortality risk.

**Interpretation:** Our results indicate that sleep oxygen saturation < 85% should be viewed as a high-risk factor for mortality in OSA and a goal for effective treatment.

OSA is typified by recurrent episodes of upper airway occlusion during sleep cycles.^1^ These episodes are implicated in a cascade of physiologic alterations, such as sympathetic nervous system activation, pronounced fluctuations in intrathoracic pressure, and intermittent hypoxemia. These physiologic disruptions are postulated to be contributory factors to comorbidities observed in patients with OSA, including hypertension, cardiovascular and cerebrovascular pathologic features, as well as metabolic dysregulation.^2^, ^3^, ^4^, ^5^ Both animal models and human clinical studies have verified that episodes of intermittent hypoxemia serve as a potent catalyst for the onset of systemic hypertension.^6^ Studies have shown that it is the systolic BP during sleep that is responsible for the adverse cardiovascular consequences of hypertension in healthy people,^7^ as well as patients with OSA.^8^ Data from Lavie et al^9^ show that mortality from OSA may be mediated through its risk for hypertension. The Sleep Heart Health Study (SHHS) was a prospective cohort study formulated to explore the risk factors for development of cardiovascular disease in OSA, diagnosed via unaccompanied home sleep apnea test with inclusion of electroencephalography.^1^^,^^10^ This included participants who were 40 years of age or older with no history of sleep apnea treatment. We used these data to compare the association of apnea-hypopnea index (AHI) and amount of sleep hypoxemia with the risk of all-cause mortality

## Study Design and Methods

Home sleep apnea tests in the SHHS were carried out using the Compumedics P Series System with monitoring of electroencephalography, electrooculography, electrocardiography, chin electromyography, thoracic and abdominal excursion by inductance plethysmography, airflow via thermistor, pulse oximetry, and body position. The baseline examination was conducted between 1995 and 1998, treatment status was determined between 1998 and 2001, and all-cause mortality was tracked until 2010. More information about the study is available in the original publication of the SHHS.^1^^,^^10^

### Variable Definitions

The cardiovascular score was adjusted for the cardiovascular risk factors at baseline as one variable to reduce the complexity of the model. This variable was calculated by adding one point for each of the following: pacemaker, heart failure, myocardial infarction, hypertension, angina, coronary angioplasty, coronary artery bypass grafting, and other heart surgery. Those treated for OSA with positive airway pressure, a mandibular-advancing oral appliance, a combination of both, or other treatment methods were designated as treated, and otherwise were designated as untreated. The AHI scoring criteria used in this analysis followed rule 1A recommended by the American Academy of Sleep Medicine (ie, apneas plus hypopneas with ≥ 30% flow reduction and ≥ 3% oxygen desaturation). The cutoffs used for mild, moderate, and severe OSA also were those recommended by the academy. Two variables were used to assess the impact of sleep hypoxemia: (1) the percentage of sleep time with oxygen saturation < 85% (PERC85) and (2) the duration of sleep (in minutes) with oxygen saturation < 85% (MIN85). Although the oxygen saturation cutoff of 88% is the most widely used in clinical practice, it was not available in this data set. Therefore, the 85% cutoff was used in our analysis as the most likely to provide a practical level of mortality significance among the available cutoffs. The thresholds of PERC85 and MIN85 categories were assigned to the values that have the most clinical relevance and where enough sample size was available for analysis.

### Statistical Analysis

The χ^2^ test and analysis of variance were used to assess statistical differences among groups for categorical and continuous variables, respectively. We performed multivariable logistic regression analyses with all-cause mortality (vital vs nonvital) and adjusted them for age, sex, BMI, pack-years of smoking, cardiovascular score at baseline, and treatment status. A second model was adjusted further for baseline history of COPD, chronic bronchitis, and emphysema. We compared the association of (1) the AHI, (2) PERC85, and (3) MIN85 with the risk of all-cause mortality. In the primary analyses, these variables were used as categorical to produce clinically meaningful outcomes. A secondary analysis was conducted using the continuous variables. ORs and 95% CIs were estimated, and the α error value was set at 0.05. All analyses were conducted using the statistical software package SAS version 9.4 (SAS Institute). Because all patient data were completely de-identified, additional institutional review board approval was waived.

## Results

**Figure 1:**
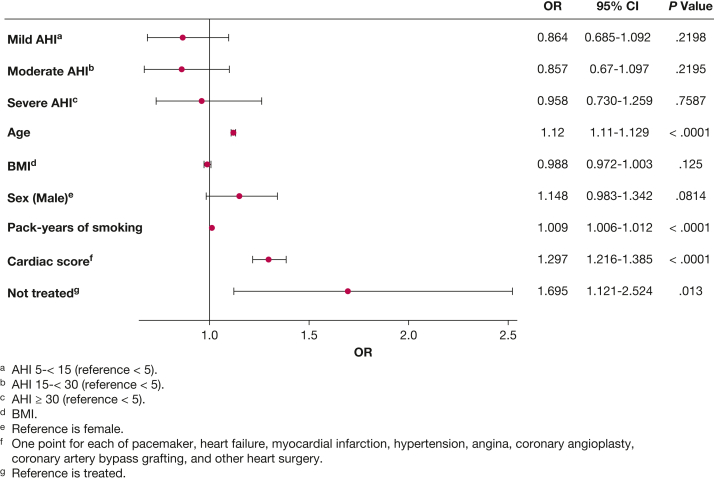
Forest plot showing mortality risk for percentage sleep with oxygen saturation < 85%. AHI = apnea-hypopnea index.

**Figure 2:**
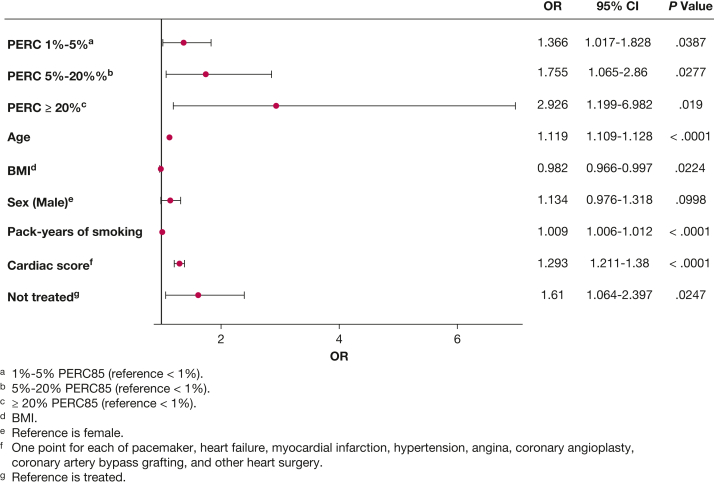
Forest plot showing mortality risk for PERC85 (minutes). PERC = percentage of sleep time with oxygen saturation of; PERC85 = percentage of sleep time with oxygen saturation < 85%.

**Figure 3:**
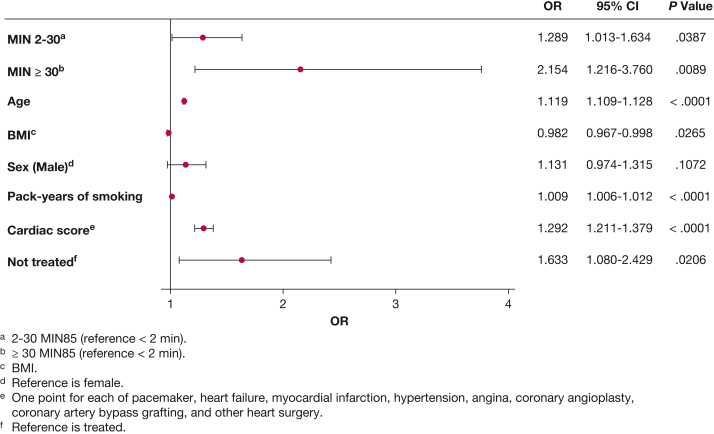
Forest plot showing apnea-hypopnea index and treatment mortality risk. MIN = duration of sleep with oxygen saturation of; MIN85 = duration of sleep with oxygen saturation < 85%.

A total of 5,804 patients were analyzed with a mean ± SD age of 63.1 ± 11.2 years, and 47.6% of patients were male. The characteristics of the total sample and based on PERC85 categories are demonstrated in Table 1. The analysis showed that PERC85 was associated with an increased risk of death (OR, 1.03; 95% CI, 1.01-1.05; *P = .*003). Patients with PERC85 of (1) 1% to 5%, (2) 5% to 20%, and (3) > 20% showed progressively higher risks compared with those with PERC85 of < 1% (OR, 1.37 [95% CI, 1.02-1.83], 1.76 [95% CI, 1.07-2.86], and 2.93 [95% CI, 1.20-6.98], respectively; *P < .*05 for all) (Fig 1). Similarly, MIN85 predicted all-cause mortality (OR, 1.01 [95% CI, 1.00-1.01]; *P = .*009). Participants with 2 to 30 min and > 30 min of MIN85 showed higher likelihoods of death vs those with < 2 min of MIN85 (OR, 1.29 [95% CI, 1.01-1.63] and 2.15 [95% CI, 1.22-3.76], respectively; *P* < .05) (Fig 2). However, AHI was not associated with an increased risk of mortality (OR, 1.00 [95% CI, 0.99-1.01]; *P* = .15), even as a severity variable (mild: OR, 0.86 [95% CI, 0.69-1.09]; moderate: OR, 0.86 [95% CI, 0.67-1.10]; severe: OR, 0.96 [95% CI, 0.73-1.26]; *P* > .05 for all) (Fig 3). Risk of death was increased significantly in those who were not treated for OSA with CPAP, oral appliances, or other treatment (OR, 1.70 [95% CI, 1.12-2.52]; *P = .*013) (Fig 3). After further adjusting for baseline COPD, chronic bronchitis, and emphysema in all previously mentioned models, PERC85 and MIN85 remained associated significantly with the risk of all-cause mortality (*P < .*05 for all categories).

**Table 1:** Sample Characteristics Based on Sleep Percentage With Oxygen Saturation Below 85%

Variable	PERC85					*P* Value^a^
	< 1% (n = 5,351)	1%-5% (n = 317)	5%-20% (n = 104)	≥ 20% (n = 32)	Total (N = 5,804)	
Vital						< .0001
No	1,155 (19.91)	102 (1.76)	35 (0.60)	13 (0.22)	1,305 (22.49)	
Yes	4,195 (72.30)	214 (3.69)	69 (1.19)	19 (0.33)	4,497 (77.51)	
AHI						< .0001
< 5	1,003 (17.28)	2 (0.03)	2 (0.03)	0 (0.00)	1,007 (17.35)	
5 to < 15	2,193 (37.78)	21 (0.36)	6 (0.10)	3 (0.05)	2,223 (38.30)	
15 to < 30	1,470 (25.33)	99 (1.71)	12 (0.21)	4 (0.07)	1,585 (27.31)	
≥ 30	685 (11.80)	195 (3.36)	84 (1.45)	25 (0.43)	989 (17.04)	
Treatment status						< .0001
Treated	5,210 (89.77)	291 (5.01)	91 (1.57)	26 (0.45)	5,618 (96.80)	
Untreated	141 (2.43)	26 (0.45)	13 (0.22)	6 (0.10)	186 (3.20)	
Sex						< .0001
Male	2,490 (42.90)	188 (3.24)	66 (1.14)	21 (0.36)	2,765 (47.64)	
Female	2,861 (49.29)	129 (2.22)	38 (0.65)	11 (0.19)	3,039 (52.36)	
Age, y	62.94 ± 11.22	66.10 ± 10.79	64.47 ± 11.26	61.84 ± 11.81	63.13 ± 11.22	< .0001
BMI, kg/m^2^	27.83 ± 4.82	31.67 ± 6.04	32.78 ± 6.92	33.48 ± 7.31	28.16 ± 5.09	< .0001
Cardiovascular index^b^	0.70 ± 1.02	0.91 ± 1.06	1.00 ± 1.19	1.13 ± 1.39	0.72 ± 1.03	< .0001
Smoking history, pack-years	13.13 ± 20.90	20.35 ± 30.19	15.70 ± 23.68	23.57 ± 27.93	13.63 ± 21.68	< .0001
History of COPD						.1114
No	5,206 (90.59)	305 (5.31)	99 (1.72)	31 (0.54)	5,641 (98.16)	
Yes	57 (0.99)	2 (0.03)	3 (0.05)	0 (0.00)	62 (1.08)	
Do not know	38 (0.66)	5 (0.09)	0 (0.00)	1 (0.02)	44 (0.77)	
History of chronic bronchitis						.0788
No	4,966 (86.08)	285 (4.94)	96 (1.66)	27 (0.47)	5,374 (93.15)	
Yes	281 (4.87)	25 (0.43)	6 (0.10)	3 (0.05)	315 (5.46)	
Do not know	74 (1.28)	3 (0.05)	1 (0.02)	2 (0.03)	80 (1.39)	
History of emphysema						.0247
No	5,186 (89.89)	299 (5.18)	96 (1.66)	31 (0.54)	5,612 (97.28)	
Yes	112 (1.94)	12 (0.21)	7 (0.12)	1 (0.02)	132 (2.29)	
Do not know	23 (0.40)	2 (0.03)	0 (0.00)	0 (0.00)	25 (0.43)	

Data are presented as No. (%) or mean ± SD unless otherwise indicated. AHI = apnea-hypopnea index; PERC85 = percentage of sleep time with oxygen saturation < 85%.

a χ^2^ test for categorical variables and analysis of variance test for continuous variables.

b One point for each of pacemaker, heart failure, myocardial infarction, hypertension, angina, coronary angioplasty, coronary artery bypass grafting, and other heart surgery.

## Discussion

OSA is correlated with an increased likelihood of hypertension developing, primarily because of the role of intermittent hypoxemia as the central contributing factor.^11^^,^^12^ In this study, we attempted to elucidate the relationship between various parameters—specifically, AHI, PERC85, and MIN85—and all-cause mortality. The severity of this condition usually is categorized based on AHI. Our findings suggest that sleep hypoxemia is a significant parameter to estimate risk of death in those with OSA and that AHI by itself may not correlate with mortality risk in the same way as apnea index alone.^13^

### Limitations of AHI as a Predictor

AHI does not account for the length of apnea and hypopnea episodes, making it unreasonable to equate events of differing durations, such as 10 s vs 30 s or 60 s, in terms of their physiologic impact. The usual sleep study report generated using the AHI falls short in quantifying MIN85. This may be of considerable importance, because variations in oxygen levels can have repercussions on other organ systems. Therefore, it is advisable that the magnitude of hypoxemia, including MIN85, be included explicitly and scrutinized in both polysomnography and home sleep apnea test reports for a more comprehensive assessment. Our multivariable logistic regression analysis, after adjusting for age, sex, BMI, pack-years of smoking, treatment status, and cardiovascular score, revealed that AHI lacks significant predictive power for all-cause mortality. This was evidenced by ORs of between 0.86 and 0.95 and corresponding *P* values exceeding .2, rendering them statistically nonsignificant. Prior studies have shown that the apnea index, without inclusion of hypopneas, is associated with increased mortality,^13^ with an apnea index of > 20 apneas/h of sleep. It is possible that the inclusion of hypopneas with 3% oxygen desaturation may have diluted the value of AHI with events that had less depth and duration of hypoxemia, and possibly less impact on sleep BP. Those with extreme obesity have been shown to have a very high hypopnea to apnea ratio with abundance of hypopneas, but paucity of apneas.^14^ The central AHI was shown to be correlated with mortality in those with ischemic cardiomyopathy and left ventricular ejection fraction of < 35%,^15^ but this may be a reflection on the propensity of the failing heart to more central apnea and Hunter-Cheyne-Stokes breathing. Although mortality resulting from heart failure has decreased over the past 10 years, an increase in mortality resulting from sleep apnea and combined heart failure and sleep apnea has occurred.^16^ An increased risk of both stroke and mortality has been reported with sleep apnea, and the risk increased with severity of sleep apnea by AHI criteria.^17^ Limitations with the SHHS database include the lack of clarity concerning treatment information, including efficacy and adherence. This may have introduced errors around the severity and duration of OSA experienced by patients and the potential impact on overall hypoxemia metrics.

### Hypoxemic Time with Oxygen Saturation Determined by Pulse Oximetry of < 85%

In contrast to AHI, the PERC85 emerged as a statistically significant and robust predictor of mortality, with ORs ranging from 1.29 to 2.93. In the context of sleep disorders, evidence suggests that it is hypoxemia—rather than the AHI—that demonstrates a correlation with excessive daytime sleepiness.^18^^,^^19^ This relationship holds true for sleepiness metrics ascertained through both subjective self-reports and objective measurements, highlighting the specific importance of addressing hypoxemia in patients experiencing daytime fatigue. These parameters remain significant even after adjusting for confounding variables. Sleep hypoxemia (like sleep BP) seems to be an independent predictor of mortality in various patient populations. Survival in patients with COPD with daytime arterial oxygen tension of > 60 mm Hg has been shown to be worse with sleep hypoxemia.^20^

### Clinical Implications

Other studies have shown the importance of hypoxemia in cardiovascular disease. Gottlieb et al^21^ reported that in patients with heart failure and OSA, hypoxemia seems to be a critical element influencing the detrimental effects of sleep abnormalities on hemodynamic stress. Accordingly, averting hypoxemia may be crucial for improving outcomes in this patient population. Our results imply that clinicians should look beyond AHI when evaluating the risk profile of patients with OSA. Sleep oxygen saturation levels of ≤ 60% have been identified as posing a dual risk: they are associated with a higher likelihood of cardiovascular disease and an increased propensity for ventricular arrhythmias.^22^ Sustained hypoxemia with oxygen saturation levels between 80% and 85% can occur in conditions such as rapid altitude changes or chronic lung disease during sleep, but this is distinct from the intermittent hypoxemia seen in OSA, primarily because of the cyclical re-oxygenation patterns and increased sympathetic nervous system activity with OSA. These cycles resemble ischemia-reperfusion injury and contribute to elevated levels of reactive oxygen species and oxidative stress.^23^ Our study suggests that oxygen saturation levels < 85% during sleep seem to be a more reliable indicator of mortality risk than AHI, perhaps because of a combination of hypoxemia duration or depth and the hemodynamic consequences of OSA. Therefore, the inclusion of this parameter in clinical practice may afford clinicians and patients with valuable information regarding the advantages of adequate treatment. It is possible that overnight sleep oximetry may identify those patients with OSA at greatest risk of adverse outcome and may facilitate their evaluation with sleep studies and adequate treatment.

### Comparison With Existing Literature

Our findings are congruent with prior studies that have indicated nocturnal hypoxemia as a concern in respiratory diseases.^24^ Azarbarzin et al^25^ studied the concept of hypoxic burden, which can be obtained readily from nocturnal sleep studies, in the SHHS and the Osteoporotic Fractures in Men Study (MrOS), but found significant correlation only with all-cause mortality in the MrOs study with men age of 76.3 ± 10.9 years, although correlation with cardiovascular disease mortality was seen in both. They excluded patients in the SHHS who were missing cardiovascular disease mortality status, whereas our study concentrated on all-cause mortality. Also, the US Preventive Services Task Force reported that existing evidence is inadequate to evaluate the tradeoff between the current advantages and disadvantages of screening for OSA in the adult population at large.^26^ However, unlike previous studies that mostly focused on AHI as a singular parameter, our study emphasized the importance of sleep hypoxemia. Punjabi et al^27^ looked at mortality in sleep-disordered breathing, but that study did not adjust for treatment status, as ours did. They did find an increased mortality risk only with AHI of ≥ 30 and in men younger than 70 years with duration of sleep with oxygen saturation < 90% (TST90). TST90 was not associated with greater mortality risk in men older than 70 years or women at any age. Smagula et al^28^ looked at mortality risk in older men with AHI of ≥ 30 in the MrOS sleep study. They found > 1% duration of sleep < 80% to be independently predictive of mortality, but not ≥ 10% TST90.

### Study Limitations

It is essential to acknowledge that the study’s design does not establish causality, but rather an association. Most importantly, oxygen saturation of < 85% encompasses not only the intermittent hypoxemia of OSA, but also sustained hypoxemia resulting from heart or lung disease or hypoventilation. Furthermore, the study did not consider possible influences of medication, lifestyle, or other comorbidities. The age-restricted cohort also may limit the generalizability of the findings. The limitations of the SHHS are notable because the AHI reported is based on listing the number of apneas and hypopneas per hour of sleep, with airflow estimated by use of oronasal thermistors without nasal pressure monitoring. However, electroencephalography monitoring of sleep was performed. Hypopneas were scored according to current guidelines with 3% oxygen desaturation or arousal. An oxygen saturation cutoff point of 88% was not available in the database.

### Future Directions

Further research should validate these findings across different populations and age groups. Prospective longitudinal studies also are needed to explore causality and to delve into the underlying mechanisms that link sleep hypoxemia to mortality, including sleep BP with 48-h ambulatory BP monitoring.

## Interpretation

Sleep hypoxemia with oxygen saturation of < 85% is an underrecognized but significant predictor of all-cause mortality in patients with OSA. This may be because of the cumulative effects of intermittent hypoxemia resulting from OSA or because of sustained hypoxemia related to obesity-hypoventilation syndrome, heart failure, or intrinsic lung disease. Although the intermittent hypoxemia of OSA is related directly to systemic hypertension^29^^,^^30^ and left ventricular disease, sustained hypoxemia may lead to pulmonary hypertension and right ventricular dysfunction.^31^

This may have contributed to the increased mortality risk in this study, because patients with obesity-hypoventilation syndrome and overlap syndrome were not excluded. Nevertheless, clinicians are advised to incorporate oxygen saturation of < 85% into their diagnostic and prognostic evaluations to provide a more comprehensive risk assessment. Future research should aim to substantiate these findings and to elucidate the causal mechanisms at play.

## Funding/Support

The authors have reported to *CHEST Pulmonary* that no funding was received for this study.

## Financial/Nonfinancial Disclosures

None declared.
